# Research Progress of MRI-based Radiomics in Rectal Cancer

**DOI:** 10.2174/0115734056450198260207200013

**Published:** 2026-02-17

**Authors:** Liuping Zhu, Yingjie Shao, Wendong Gu

**Affiliations:** 1 Department of Radiation Oncology, Changzhou First People's Hospital, Changzhou Medical Center, Nanjing Medical University, Nanjing, Changzhou 213003, China

**Keywords:** Magnetic resonance imaging, Radiomics, Rectal cancer, Malignant tumors, Tumor heterogeneity, Tumor-node-metastasis

## Abstract

Rectal cancer (RC), one of the most common malignant tumors, has a high incidence rate and mortality rate worldwide. Radiomics turns medical images into high-dimensional mineable data through high-throughput extraction algorithms, where the methods include filter-based algorithms and texture analysis. All these features are then combined with machine learning or deep learning algorithms to provide objective evidence to facilitate accurate diagnosis, radiation staging, radiotherapy planning, or prognosis prediction. Multi-parametric magnetic resonance imaging has been considered as one of the best modalities for performing radiomics analysis on rectal cancer because it can capture most features about tumor heterogeneity and micro-environment information. In the past few years, magnetic resonance imaging (MRI)-based radiomics has shown great promise in a variety of fields, including tumor-node-metastasis staging, monitoring pathological high-risk factors, predicting genetic markers, neoadjuvant therapy response evaluation, and prognostic survival analysis in rectal cancer. In this paper, we provide an overview of the current state-of-the-art on MRI radiomics for rectal cancer and present a comparison between the available methods of feature extraction, and provide a critical discussion of current issues and possible developments that might be pursued in future research on this topic.

## 1 INTRODUCTION

Colorectal cancer (CRC) is considered a common malignant tumor and ranks third in the global cancer incidence rates [1]. Rectal cancer (RC) accounts for approximately 38% of CRC cases [2], and achieving accurate diagnosis and meticulous formulating a treatment plan is crucial for improving patient prognosis. Magnetic resonance imaging (MRI) is widely considered the imaging modality of choice for local staging and restaging, owing to its superior soft tissue contrast resolution [3]. Additionally, MRI plays an indispensable role in assessing therapeutic response, monitoring disease progression, and detecting postoperative local recurrences. Nevertheless, conventional MRI is mainly based on the morphology evaluation with the subjectivity of clinical experience, which cannot fully describe the tumor heterogeneity and biology. Traditional T2-weighted imaging (T2WI) primarily evaluates morphological features of tumors, includingsize,delineation,andsignalintensity;butithas limitations in the ability to identify subtle microlesions at an earlier stage, lymph node micrometastasis, or other molecular characteristics. While functional techniques such as diffusion-weighted imaging (DWI) and dynamic contrast-enhanced (DCE) MRI offer hemodynamic insights, they are hindered by complex parameter quantification and variation in the protocols [4]. In 2012, Professor Lambin introduced the concept of radiomics [5]. It is a new way for medical imaging big data analysis. Radiomics uses computer algorithm technology to extract large amounts of quantifiable information invisible to the naked eye from medical images. By establishing the relationship of these numerical characteristics to medical results, predictive or assessment models may be developed, thereby facilitating a more objective appraisal of the tumor’s molecular characteristics, treatment efficacy, and prognostic outlook. This approach provides physicians with new tools to achieve personalized care.

### 1.1 Radiomics Workflow

The general process of the radiomics workflow mainly includes the following steps.

#### 1.1.1 Data Acquisition and Image Segmentation

The high-quality MRI data is the key to the analysis, requiring consistent scanning parameters to reduce systematic bias. ROIs for tumors were obtained using manual, semi-automatic, or fully automatic methods [6]. Popular software packages are the 3D Slicer and the ITKS-NAP.

#### 1.1.2 Feature Extraction and Selection

This phase involves deriving high-dimensional quantitative data from the ROI, including shape features (quantifying geometry), first-order statistics (intensity distribution), texture features (*e.g*., gray-level co-occurrence matrix [GLCM], gray-level run-length matrix [GLRLM]), and higher-order features (*e.g*., wavelet transforms, Laplacian of Gaussian [LoG]) that collectively characterize complex tissue patterns. Subsequently, we apply dimensionality reduction methods such as least absolute shrinkage and selection operator (LASSO) and principal component analysis (PCA) to retain only the most effective features [7, 8].

#### 1.1.3 Model Construction and Validation

This phase explains predictive models are established using machine learning algorithms (*e.g*., support vector machine [SVM], random forest [RF]) or deep learning frameworks. Model stability and generalizability are assessed through cross-validation or validation with independent cohorts [5].

In contrast to computed tomography (CT) radiomics using Hounsfield Units, MRI radiomics can exploit multiparametric physiological information representing different proton environments in water molecules. Among magnetic resonance modalities, multi-parametric MRI (mp-MRI) is the preferred method for radiomic feature extraction in RC. While T1-weighted imaging (T1WI) and T2WI provide detailed anatomical texture, functional sequences contain other biologically meaningful features: DWI reflects cellularity due to diffusion-restricted movement of water molecules, and DCE-MRI provides information about vascular permeability by means of contrast agent kinetics [3, 4]. Consequently, the combination of anatomical (T1WI/T2WI) and functional (DWI/DCE) signatures results in the best possible description of tumor heterogeneity. Figure **1** presents a typical schematic illustration of the MRI-based radiomics workflow, spanning from data acquisition to specific clinical applications in rectal cancer.

## 2 CLINICAL APPLICATIONS OF MRI RADIOMICS IN RC

Since its inception, methods for this task have evolved considerably: early works mostly extracted handcrafted features of conventional T2WI sequences, while lately the research is more focused on multiparametric fusion, delta radiomics, as well as DL based fusion approaches [9-11]. In Table **1**, we give an overview of key developments and representative studies in chronological order.

### 2.1 Tumor-node-metastasis (TNM) Staging Determination

Surgery is preferred in early-stage RC, and neoadjuvant chemoradiotherapy (nCRT) has been commonly performed to treat patients with locally advanced disease or when sphincter preservation is difficult, which preceded total mesorectal excision (TME) and thus improved chances for anal preservation. Hence, precise assessment of tumor staging and extent of anal preservation is very important to plan further treatment strategies. Currently, high-resolution MRI represents the preferred imaging modality for RC staging, but accurate discrimination of T2 *vs.* T3 lesions still poses a challenge because of the morphological overlap between fibrosis and inflammation. To overcome such a limitation, MRI-based radiomics has shown promise in improving the accuracy of the TNM stage. Here, we summarize 13 relevant studies which were searched by using PubMed and Web of Science (Table **2**).

Radiomics can significantly increase the accuracy for T-staging, which is based on tumor extension (depth) and local spread (invasion). In an early study, Ma *et al.* [11] extracted a total of 1,029 features based only on high-resolution T2WI sequence MRI and achieved an AUC of 0.809 by using SVM-based models. The following studies have greatly improved the prediction effect thanks to their improvements in methods: Hou *et al.* [10] used 3D super-resolution *via* deep learning which achieved an AUC value of 0.869 to discriminate between T1/2 and T3/4 stages; Fan *et al.* [12] used the feature extracted by deep transfer learning along with clinic factors, thus improving AUC by 0.920 for classification between stage T2 and T3. Furthermore, Deng *et al.* [13] first introduced the features extracted from mesorectal fat into the analysis and showed excellent performance in pre-operative classification between T2 and T3 stages for RC diagnosis. Additionally, Ao *et al.* [14] proposed a novel notion called “tumor microenvironment habitat,” and partitioned the tumor into three functionally distinct sub-regions using the k-means clustering technique, where the tumor microenvironment C model provides better prediction results (AUC, 0.817–0.981).

Lymph node metastasis (LNM) has been considered the most common pattern of metastasis for CRC, and its occurrence rate of metastasis could be up to 52% and it has been identified as an important high-risk marker of local recurrence, and also for distant metastases after CRC surgery [15]. Conventional MRI evaluation is mainly based on morphologic features, dimensional criteria, and signal intensity for evaluating the LNM. However, as the anatomic position and node size are variable among individuals, these assessments have limited specificity, although it has low sensitivity for the detection of lymph node micrometastases. MRI-based radiomics features can also improve the predictability of LNM, as Li *et al.* [16], they used a multi-objective immune algorithm to choose out 12 key features, thus constructing a combined model that achieved an AUC of 0.94 with a sensitivity of 92.2%, significantly outperforming the subjective ratings by radiologists (sensitivity 72.1%). Fang *et al.* [9] introduced a novel idea, termed “Delta-radiomics”, where changes of ADC feature pre- and post-neoadjuvant therapy were used for the prediction of ypN status, resulting in an AUC of 0.895 and showed a significant association to 5-year DFS within the ypT0-2 subgroup (*P* = 0.030). Additionally, Niu *et al.* [17] compared the results of CT and MRI-based models, demonstrating that the MRI multi-parameter model (T2WI + contrast-enhanced T1-weighted imaging [CE-T1WI]) yielded a superior AUC of 0.785 than CT (0.721), with the Clinical-IMG model, which integrated clinical variables, further enhancing the AUC to 0.947. Furthermore, a multi-parameter MRI nomogram was established by Zheng *et al.* [18], which achieved a 0.880 AUC on an external test set and showed statistically significant improvement over two other competing models, demonstrating substantial potential value as a preoperative prediction of LNM in RC.

Distant metastasis is an important poor-prognosis factor for CRC patients, and the liver is the most common site of distant metastasis. Thus, accurate prognosis of liver metastasis risk in CRC can facilitate the early detection of high-risk patients and to initiate more aggressive treatment plans, eventually leading to better patient survival. Shu *et al.* [19] developed a prediction model based on the feature derived from T2WI with an AUC of 0.857 in predicting synchronous liver metastasis (SLM) prediction, while adding clinic variables in the nomogram model increased the AUC value up to 0.921. The clinical-radiomics model developed by Liu *et al.* [20] had a sensitivity of 72.2% and a specificity of 94.4% for synchronous distant metastasis (SDM) prediction, with a significantly improved predictive value of their nomogram for clinical decision making. Additionally, there are some recent studies that try to relate imaging features with tumor biology through integrating molecular markers and pathological attributes. For instance, Ma *et al.* [21] combined Kirsten rat sarcoma viral oncogene homolog (KRAS) mutation status with radiomics-derived features, resulting in a composite model that achieved an AUC of 0.842, thus confirming that the mutation genes play a contributing part in optimizing liver metastasis prediction for RC. In a recent analysis, Zhao *et al.* [22] introduced a composite model integrating the tumor-stroma ratio derived from colonoscopic biopsy specimens with radiomics features, proving its practical simplicity and efficacy in stratifying distant metastasis risk.

Collectively, recent data suggest that although T2WI is still the gold standard for staging, single-sequence models are generally challenged to discriminate the thin boundary between stage T2 *vs.* stage T3. Recent reports show a clear advantage in combining DL and radiomics over conventional ML approaches, raising AUCs from ~0.80 to >0.90 [10, 12]. However, LNM prediction is still difficult in comparison with T-staging, possibly because of a very heterogenous nodal involvement. Future models incorporating mesorectal fat features and peritumoral habitats will be required to increase sensitivity for microscopic nodal disease [13, 14].

### 2.2 Prediction of High-risk Clinical Pathological Factors

Inclusion of various clinicopathological variables in a risk classification system allows us to devise more aggressive treatment regimens for those patients, thus reducing the risk for local recurrences and distant metastases. MRI-based radiomics have shown promising predictive ability for important histopathologic parameters of RC in the past few years, including extramural vascular invasion (EMVI), peripheral fat infiltration (PFI), perineural invasion (PNI), lymphovascular invasion (LVI), circumferential resection margin (CRM), and tumor budding (TB). In this section, we summarize findings from 12 studies performed in the last five years that have used MRI radiomics with a specific aim of predicting some histopathological factors in RC (Table **3**).

Radiomics was demonstrated as a means for improving the detection rate of EMVI based on tumor edge characteristics. For instance, Zhao *et al.* [23] and Shu *et al.* [24] performed radiomics analyses using synthetic MRI relaxation maps and multi-parameter MRI features, respectively, for predicting the EMVI preoperatively in RC with good diagnostic performances. The nomogram incorporating the clinicoradiomic factors was developed by Wang *et al.* [25] and Wang *et al.* [26], demonstrated superior predictive capability for preoperative identification of PFI and PNI, respectively, offering important guidance in making clinical decisions. Accurate and non-invasive prediction of LVI remains essential for prognostic management in RC patients. Xu *et al.* [27] constructed a combined model using clinic parameters in combination with MR-derived radiomic features from the tumor (intratumoral) and extra-tumor (peritumoral) areas, where the feature combination of the intratumoral region and a 3-mm peritumoral region on T2WI was chosen as the best model, which achieves an AUC value of 0.913. Similarly, Ning *et al.* [28] created a clinical-radiomics combined model to predict LVI with an AUC of 0.90, 0.88, and 0.88 in the training, internal validation, and external validation cohorts, respectively. Moreover, Liang *et al.* [29] performed a comparison study between traditional MRI analysis and MRI-derived radiomics to determine CRM in RC, suggesting that radiomics modeling for RC and the mesorectal fascia had similar performance and that an integrated nomogram model was more accurate than either alone. Interestingly, one of the latest studies [30] has shown that an MRI-based T2WI sequence-derived radiomic model performed better than human reading at predicting cellular mucin after nCRT for RC, thereby assisting in differentiating residual adenocarcinoma from acellular mucin protein and offering a useful guide for therapeutic planning.

Tumor budding has been recognized as an important prognostic factor in RC, and the diagnosis of tumor budding was defined based on histopathological examination. However, several recent studies demonstrated that MRI radiomics could provide great help for noninvasive and preoperative prediction of tumor budding grade in RC [31-34]. For example, Li *et al.* [31] selected five image-based key features that were significantly related to tumor budding grade from multi-sequence MRI, including T2WI, CE-T1WI, and DWI. The resulting multi-sequence radiomics model demonstrated superior predictive performance, attaining an AUC of 0.796 and an accuracy of 81.2% in the validation cohort. Additionally, a recent study [32] employed multi-parameter MRI radiomics combined with a three-dimensional visual transformer-based deep learning model for effective and non-invasive prediction of tumor budding grading in RC patients, thus providing meaningful information for personalized treatment planning and prognostic evaluation.

In conclusion, all the reviewed studies confirm how it’s possible to use radiomics as a non-invasive virtual biopsy in order to identify aggressive pathological features. It emerges from these studies how the addition of “peritumoral” (*i.e*., around the tumor) radiomic analysis may provide an added benefit, especially in the prediction of invasiveness (*e.g*., LVI and TB) [27, 29]. Moving forward, it is necessary to standardize the margin of the peritumoral region (*e.g*., 3mm *vs.* 5mm) for comparing results across different institutions [27, 35].

### 2.3 Prediction of Genetic Markers

Imaging phenotypes extracted from radiomics studies were related to the tumor’s genetic profile: this is called “radiogenomics”, which is widely used for tackling tumor heterogeneity, immune response prediction, or tumor evolution forecasting [36]. In RC patients, the detection of genetic markers-particularly KRAS expression and microsatellite instability (MSI) status-is highly relevant to predict therapeutic response after nCRT as well as prognosis. The following list shows 11 contemporary clinical studies investigating the use of MRI radiomics to predict genetic markers in RC (Table **4**).

KRAS mutations have been identified in approximately 35% to 45% of CRC, and patients with KRAS mutations generally do not respond well to anti-epidermal growth factor receptor (EGFR) therapy, including cetuximab, while the wild-type patients have a greater chance for successful treatment. Therefore, determination of KRAS gene status is especially important in order to guide further choice of anti-tumor therapy [37-39].

Several works have proved the feasibility of applying MRI radiomics to predict KRAS expression without invasiveness in RC patients. For instance, Cui *et al.* [40] built a radiomics model of the T2WI feature-based model showed fair prediction performance in predicting KRAS status, resulting in an AUC of 0.722 (training), 0.682 (internal validation), and 0.714 (external validation). Similarly, the feasibility of KRAS mutation prediction in RC was further corroborated by MRI radiomics models established by Guo *et al.* [41] and Zhang *et al.* [8]. Additionally, Meng *et al.* [42] developed a radiomics model based on mp-MRI, which not only evaluated KRAS-2 status (AUC of 0.651), but also assessed other biomarkers, including the percentage of Ki-67 positive tumor cells (AUC of 0.699) and HER2 (AUC of 0.696). Furthermore, Zhou *et al.* [43] retrospectively analyzed 1,886 locally advanced rectal cancer (LARC) cases and found no significant association between the presence of KRAS mutation and lower pathological complete response (pCR) rate or neoadjuvant rectal (NAR) score after neoadjuvant therapy, but correlated with lower survival and worse prognosis after nCRT. Xiang *et al.* [44] further studied the correlation of pretreatment MRI radiomics predicted KRAS status with pulmonary metastasis in LARC patients, finding that high radiomics scores correlated with more frequent lung metastases.

The origin of MSI is due to a mutation in the mismatch repair (MMR) gene caused by dysfunction of the MMR system and/or MutL Homolog 1 (MLH1) hypomethylation, thus promoting CRC development at a faster pace [45, 46]. Over the last few years, there have been several studies that focus on building and evaluating an mp-MRI-based radiomics model for predicting MSI status among RC patients [47-52]. For instance, Cai *et al.* [47] proposed a subregional radiomics model based on mp-MRI, which surpassed traditional radiomics and clinical-radiological models in predictive accuracy, achieving AUCs of 0.86 (training), 0.72 (internal validation), and 0.59 (external testing). The subregional-clinical-radiological model integrating the clinical-radiological data and the subregional radiomics features achieved high performance with AUCs of 0.87 (training) and 0.85 (external validation). Furthermore, Yao *et al.* [51] designed and validated a multimodal deep learning model that integrated clinical data with mp-MRI as well as hematoxylin and eosin-stained pathological images. With the help of multimodal information, this model well captured the tumor heterogeneity and could predict the MSI status with a high degree of accuracy and generalizability among RC patients, with AUC values of 0.987, 0.987, and 0.974 across training, internal validation, and external validation cohorts. Additionally, Jing *et al.* [48] established four prediction models based on preoperative MRI to predict MMR status in RC patients, with the multi-sequence model having a better diagnostic performance across all datasets.

In conclusion, it appears from the current literature that there exists some disparity with respect to the predictive potency of genetic biomarkers: radiomics-based modeling seems to have better accuracy in predicting MSI status than KRAS mutation (AUC > 0.85 *vs.* ~0.70-0.80, respectively) [40, 51]. It could be because MSI tumours have specific morphological phenotypes (*e.g*., mucinous components, lymphocyte infiltration), which can be better seen with MRI than the small changes in signal pathways induced by the presence of a KRAS mutation [45]. The multimodal fusion models with the inclusion of clinical information are superior to the radiomics-only model for all tasks in this field.

### 2.4 Evaluation of Efficacy and Response to Neoadjuvant Therapy

Due to lack of classic early clinic symptoms among RC patients, about 85% of them were diagnosed with LARC disease, and due to high recurrence rates after direct resection or chemoradiation among LARC patients, the sequence of nCRT and subsequent TME is considered to be the gold standard treatment modality in this group [53]. Chemoradio-therapy before surgery results in a reduction of the tumor volume and may cause a tumor downstaging as well as reduce risk for locoregional recurrences, and improve the quality of life in patients [54]. Nevertheless, tumor responses to nCRT vary considerably among individuals; certain patients display resistance, whereas approximately 10%-30% may achieve pCR [55]. Performing TME in patients who attain pCR could result in severe postoperative complications, including an increased risk of anastomotic leakage [56]. For such cases, a “wait-and-watch” strategy is generally recommended, demonstrating favorable long-term outcomes with markedly reduced rates of recurrence and metastasis compared to non-pCR counterparts [57]. Thus, given that there is a large patient-to-patient variation with respect to the effectiveness of nCRT amongst LARC patients, accurate prediction of treatment outcome before initiation of therapy, particularly identifying pCR, is crucial in guiding the choice of personalized, precise treatment strategy.

Since Nie *et al.* [58] first proposed the application of MRI radiomics to predict pCR after NACT in 2016, an increasing number of studies have used MRI radiomics to quantitatively evaluate the effectiveness of neoadjuvant therapy for RC [7, 59-81]. After a systematic literature search on the PubMed database and the Web of Science Core Collection, we found that 41 articles were investigating the correlation between MRI radiomics and predicting treatment responses following neoadjuvant therapy in RC, of which we included 25 papers in our review (Table **5**).

For instance, Yi *et al.* [79] established a T2WI-based radiomics model that achieved high diagnostic accuracy in predicting pCR, good response (GR), and tumor downstaging, with corresponding AUCs of 0.908, 0.902, and 0.930, respectively. Considering individual variability, an increasing number of investigations have incorporated patient-specific clinical risk factors into predictive models. Cheng *et al.* [61] constructed nomograms through multivariate logistic regression by integrating radiomics features with key clinical indicators (carcinoembryonic antigen [CEA] and carbohydrate antigen 19-9 for GR prediction; CEA, post-treatment tumor length, and post-treatment thickness for pCR prediction). Their models demonstrated robust predictive performance, with AUCs of 0.918 and 0.944 in the training and validation cohorts for GR, and 0.959 and 0.912 for pCR, respectively. The potential clinical utility of these nomograms was further supported by decision curve analysis. Homsi *et al.* [64] retrospectively analyzed data from two institutions, utilizing T1WI, T2WI, and DWI sequences. Their findings indicated that pre-treatment multiparametric MRI radiomics models effectively predicted pCR in LARC patients, surpassing the predictive power of clinical parameters and CEA, although larger-scale validation remains necessary. Early radiomics research frequently relied on feature extraction from either pre- or post-treatment MRI alone, limiting the ability to capture dynamic tumor changes during therapy. To address this limitation, the concept of Delta radiomics was introduced, defined as the percentage change in radiomics features from pre- to post-nCRT MRI. Wan *et al.* [74], for example, developed prediction models using the T2WI and DWI Delta radiomics features as well as combining them together that had better ability in predicting pCR than the conventional MRI evaluation. Their integrated model obtained the following AUCs: 0.91 (training) and 0.91 (testing). It should be noted, however, that some works were devoted to predicting only pCR, while others concurrently assessed end points like CR, GR, or T downstaging that are also related to the treatment response. Xiang *et al.* [77] who developed and validated a predictive model of T downstaging (ypT0-2) after nCRT among LARC patients; the nomogram that integrated both radiomics and clinical features, was superior to the radiomics-only model (AUCs of 0.842 and 0.809 for the training cohort and testing cohort), which was able to identify patients that would be most likely to benefit from consolidation chemotherapy.

Critically, there is currently some evidence suggesting that Delta-radiomics (dynamic change of tumour heterogeneity over the course of nCRT) is generally associated with improved prediction of pCR when compared to analysis at one timepoint alone [9, 74]; however, standardization of repeat scan frequency across centers is currently limiting uptake. Moreover, even though multiparametric models are in general superior to those trained on a single sequence, T2WI is still the best performing sequence with regard to the reproducibility [82].

### 2.5 Prediction of Recurrence, Prognosis, and Survival Analysis

About 30% of RC patients are reported to experience local recurrence and distant metastasis within three years following surgical intervention [83]. The common local recurrence site is anastomotic, residual rectum, presacral fascia, perirectal soft tissue, or nearby pelvic organ(s) [84]. Radiomics facilitates clinical prognostic stratification by systematically quantifying adverse indicators such as mesorectal fascia involvement, depth of extramural tumor infiltration, extramural venous invasion, regional lymph node metastasis, and distant metastasis. Through a literature search from PubMed and Web of Science Core Collection databases, we retrieved 14 articles that used MRI-based radiomic features to predict prognosis or recurrence of RC patients (Table **6**).

Thirteen studies have been performed for the prediction of overall survival (OS), disease-free survival (DFS), 3-year recurrence-free survival, 5-year progression-free survival, and distant metastasis-free survival in RC patients [35, 85-96]. For instance, Guo *et al.* [89] investigated the relationship between intratumoral, peritumoral, and combined radiomic features with OS. Among these, the combined model exhibited superior correlation with OS, achieving AUC values of 0.954 and 0.821, surpassing models based on intratumoral or peritumoral features alone. In a recent retrospective analysis, Fu *et al.* [88] examined pre-treatment CEA-negative RC patients from three centers and established two DFS prediction models utilizing radiomics signatures (RS) derived from T2WI and DWI images. Model performance was assessed through Harrell’s concordance index (C-index), indicating that the All-Clin+Rad model, integrating RS with clinicopathological variables, demonstrated robust predictive capability with C-indices of 0.775, 0.739, and 0.822 across the respective cohorts. Similarly, Cui *et al.* [86] established a radiomics nomogram combined with clinic risk factors, including the pN stage and tumor differentiation. The discriminative ability between these models was assessed by Harrell’s C-index and time-independent receiver operating characteristic analysis. The results showed that the radiomics nomogram performed better than the clinicopathological model for predicting DFS, with C-indices of 0.780 (training cohort) and 0.803 (validation cohort), respectively. In addition, we found that those who had a higher risk of recurrence according to the nomogram benefited more from adjuvant chemotherapy than did the lower-risk group.

Furthermore, Chen *et al.* [84] showed that MRI-derived radiomics could discriminate between recurrent and non-recurrent lesions in the anastomosis area. Multi-sequence MRI seemed to significantly improve the prediction of recurrence on follow-up, yielded an AUC of 0.864, where the sensitivity and specificity for this model were found to be 81.82% and 75.86% on the validation cohort, respectively.

Taken together, radiomics has the potential to provide an effective means of long-term monitoring as it may help distinguish between recurrence and posttreatment fibrosis -a well-known blind spot for standard MRI [84]. Integration with “time-to-event” information (*e.g*., Cox proportional hazards models), rather than binary classification alone, to perform survival analysis has improved the statistical soundness in more recent works [88, 96]. Future efforts will need to include external validations within other ethnic cohorts so as to prove that such prognostic signatures are applicable worldwide.

## 3 CHALLENGES

Despite these encouraging results from the retrospective studies (AUC >0.90), there remains a substantial ‘translation gap’. Very few models have been prospectively validated, let alone translated to practice; largely because of ‘optimism bias’ intrinsic to small-sample retrospective training [97, 98]. Firstly, data standardization remains a critical concern, as radiomics features are derived from conventional imaging equipment. Inconsistencies between different scanners (manufacturer, model), image acquisition protocol, sequence, or reconstruction algorithm generate variations on the raw images; ultimately impacting the reliability of the extracted RS and hampering model generalizability. In order to overcome such problems, the creation of standardized multi-center databases is considered necessary [99-101]. Secondly, most existing research is retrospective, small-sample-size studies from a single center that are subject to potential selection bias. If more features are extracted than samples, the danger to be overfitted by a model is increased significantly. Thus, large-scale, prospective, multi-center clinical trials are required to ensure the robustness and clinical utility of these predictive models [82, 97]. Lastly, considerable heterogeneity persists in the delineation of ROI and volumes of interest (VOI) among different research groups. Although the majority of MRI-based radiomics investigations performed for RC are tumor-oriented segmented studies, some also include positive lymph nodes, peritumoral regions, and surrounding tissues. Each of these segmentation decisions contains different types of tumor information. In addition, ROI and VOI annotation mainly rely on manual work, which is labor-intensive and time-consuming, and sensitivity towards the observer variability. These methodological drawbacks unavoidably compromise reproducibility and model stability [82, 98].

## CONCLUSION

In the future, along with further developments on artificial intelligence techniques and the utilization of multimodal information, MRI-based radiomics may be regarded as a potential imaging biomarker with increasing importance for precise diagnosis and treatment of RC. In recent years, many potential applications have been explored, including TNM stage, predicting the risk of pathological factors, diagnosis, detection of genetic biomarkers, assessment of response to neo-adjuvant therapies and drug sensitivity, recurrence risk, and prognosis. To bridge this gap between basic science and clinical application, future work could be carried out along these three practical lines: Firstly, it is necessary to develop reliable and completely automated segmentation tools (*e.g*., based on nnU-Net frameworks) that are essential for eliminating the effect of inter-observer variability and streamlining the radiomics workflow. Secondly, research needs to move beyond monomodal analyses toward the integration of multiple modalities, including both radiomics as well as pathomics (digital pathology), genomics (liquid biopsy) in order to represent tumor heterogeneity at a range of spatial scales from macroscopic to microscopic levels. Last but not least, instead of individual algorithms, ultimately it would be desired to develop AI-based Clinical Decision Support Systems (CDSS) which could be directly integrated in Picture Archiving and Communication Systems (PACS), to deliver explainable visualizations, which can then be utilized by the clinician to plan individualized therapy.

## Figures and Tables

**Fig. (1):**
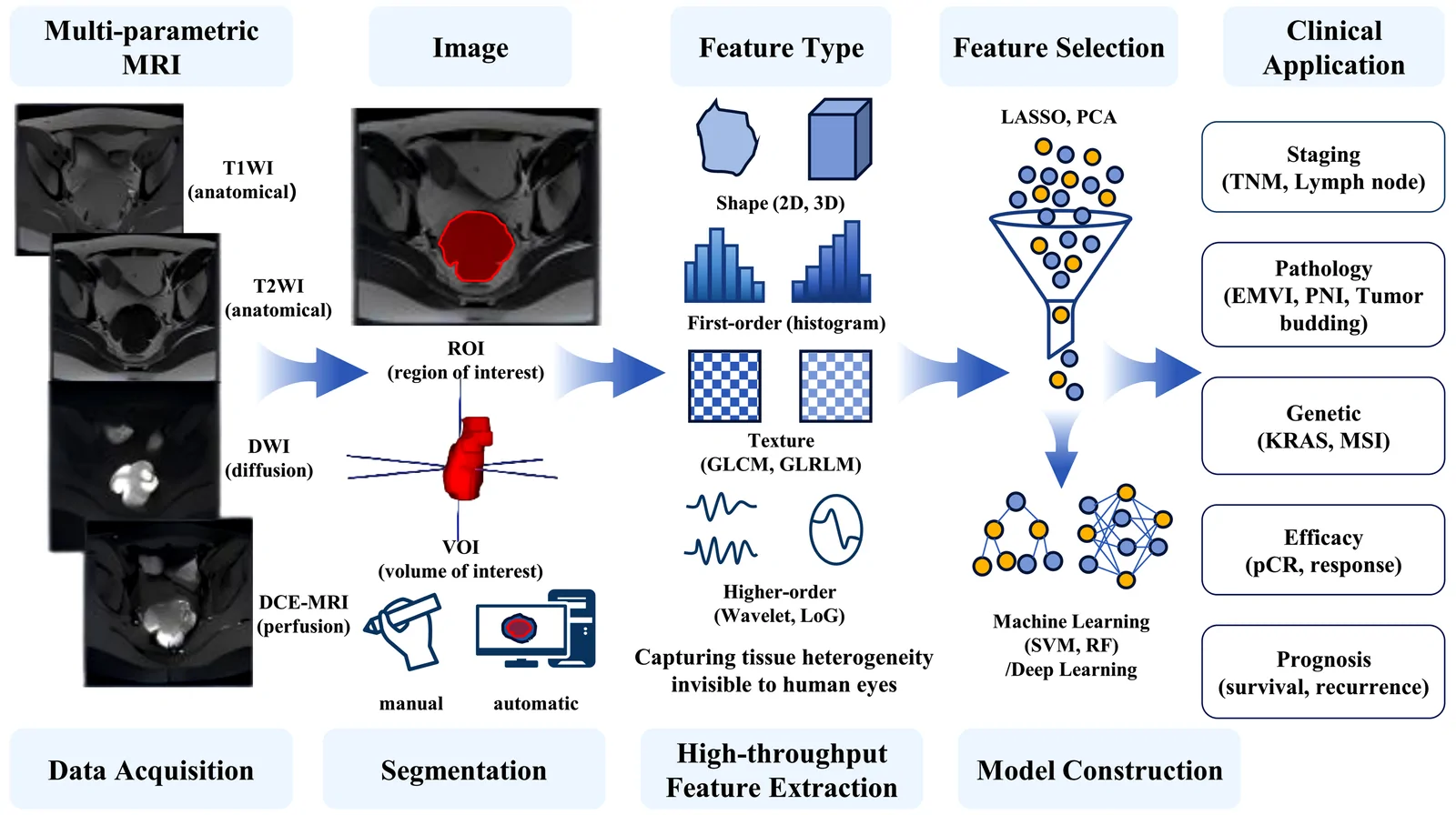
Overview of complete MRI-based radiomics workflow in rectal cancer. It illustrates the transformation of medical images into quantitative mineable data through five key steps: (1) Data Acquisition: Multi-parametric MRI displayed side-by-side, showing T1WI/T2WI (anatomical), and DWI/DCE-MRI (functional/physiological). (2) Segmentation: Delineation of Regions of Interest (ROI) or Volumes of Interest (VOI) using either manual or automatic methods. (3) High-throughput Feature Extraction: Quantification of tissue heterogeneity to Shape (geometry), First-order (histogram), Texture (*e.g*., GLCM, GLRLM), and Higher-order features (*e.g*., Wavelet, LoG). (4) Model Construction: Feature selection (*e.g*., LASSO, PCA) followed by Machine Learning (*e.g*., SVM, RF) or Deep Learning modeling. (5) Clinical Application: Application to tasks such as Staging, Pathology prediction (EMVI, PNI), Genetic profiling (KRAS, MSI), Efficacy evaluation (pCR), and Prognosis. **Abbreviations:** T1WI, T1-weighted imaging; T2WI, T2-weighted imaging; DWI, diffusion-weighted imaging; DCE, dynamic contrast-enhanced; GLCM, gray-level co-occurrence matrix; GLRLM, gray-level run length matrix; LoG, Laplacian of Gaussian; LASSO, least absolute shrinkage and selection operator; PCA, principal component analysis; SVM, support vector machine; RF, random forest; EMVI, extramural vascular invasion; PNI, perineural invasion; KRAS, Kirsten rat sarcoma viral oncogene homolog; MSI, microsatellite instability; pCR, pathological complete response.

**Table 1:** Chronological summary of representative studies and methodological evolution in rectal cancer MRI radiomics.

Time Period	Key Development / Focus Area	Representative Studies	Major Contribution / Methodology Shift	Refs.
2012	Concept Proposal	Lambin *et al.*	Proposed the fundamental concept of converting medical images into mineable quantitative data.	[5]
2016–2019	Early Application & Staging	Ma *et al.* (2019) Nie *et al.* (2016)	Pioneered mp-MRI radiomics for pCR prediction; Established baseline T2WI models for T-staging.	[11, 58]
2019–2020	Nomogram Integration	Cui *et al.* (2020) Yi *et al.* (2019)	Shifted from pure radiomics to Clinical-Radiomics Nomograms, integrating clinical factors (*e.g*., CEA) and expanding to KRAS prediction.	[40, 79]
2021–2023	Delta-radiomics	Fang *et al.* (2023) Wan *et al.* (2021)	Validated Delta-radiomics (feature changes pre- to post-treatment) to capture dynamic tumor response better than single-timepoint features.	[9, 74]
2023–2024	Deep Learning & Super-resolution	Hou *et al.* (2023) Fan *et al.* (2024)	Integrated Deep Learning (DL) and super-resolution techniques, significantly raising staging accuracy (AUC > 0.85).	[10, 12]
2025	Habitat & Multimodal Fusion	Ao *et al.* (2025) Yao *et al.* (2025)	Advanced to “Tumor Habitats” (subregional analysis) and Multimodal Fusion (integrating pathomics/genomics), enabling non-invasive microenvironment profiling.	[14, 51]

**Abbreviations:** mp-MRI, multi-parametric MRI; pCR, pathological complete response; DL, Deep Learning; AUC, area under the curve.

**Table 2:** Summary of TNM staging determination MRI radiomics-based studies.

First Author, Year	Study Design	MRI Time	Image Modality	Segmentation	Feature Selection Model	Endpoint	Refs.
Ma *et al.*, 2019	R/S	Pre	HRT2WI	ROI	LASSO	T, N	[11]
Hou *et al.*, 2023	R/S	Pre	HRT2WI, SRT2WI	VOI	LASSO	T	[10]
Fan *et al.*, 2024	R/S	Pre	T2WI	ROI, VOI	SCC, LASSO	T	[12]
Deng *et al.*, 2024	R/S	Pre	T2WI, ADC, DWI	ROI	LASSO	T	[13]
Ao *et al.*, 2025	R/M	Pre	T2WI, DWI, CE-T1WI	ROI	mRMR, LASSO	T	[14]
Li *et al.*, 2021	R/S	Pre	T2WI	ROI	IMIA	LN	[16]
Fang *et al.*, 2023	R/S	Pre, Post	T2WI, DWI, ADC	ROI	correlation analysis, RFE-LR	LN, 5y DFS	[9]
Niu *et al.*, 2023	R/S	Pre	NCE-CT, CE-CT, T2WI, CE-T1WI	ROI	PCC, RFE	LNM	[17]
Zheng *et al.*, 2024	R/M	Pre	T2WI, DWI, CE-T1WI	ROI	mRMR, LASSO	LNM	[18]
Shu *et al.*, 2019	R/S	Pre	T2WI	ROI	LASSO, PCA	SLM	[19]
Liu *et al.*, 2019	R/S	Pre	T2WI	VOI	RF	SDM	[20]
Ma *et al.*, 2024	R/S	Pre	T2WI	ROI	LASSO, multivariate Cox analysis	LM	[21]
Zhao *et al.*, 2025	R/S	Pre	T2WI	VOI	LASSO	DM, 3y DMFS	[22]

**Abbreviations:** R, retrospective study; S, single-centre study; M, multicentre study; Pre, pretreatment; Post, post-treatment; T2WI, T2-weighted imaging; HRT2WI, high-resolution T2-weighted imaging; SRT2WI, super-resolution T2-weighted imaging; ADC, apparent diffusion coefficient; DWI, diffusion-weighted imaging; CE-T1WI, contrast-enhanced T1-weighted imaging; CE-CT, contrast-enhanced computed tomography; NCE-CT, non-contrast enhanced computed tomography; ROI, region of interest; VOI, volume of interest; LASSO, least absolute shrinkage and selection operator; mRMR, minimum redundancy maximum relevance; IMIA, iterative multi-objective immune algorithm; RFE, recursive feature elimination; RFE-LR, recursive feature elimination with logistic regression classifier; SCC, spearman correlation coefficient; PCC, pearson correlation coefficient; PCA, principal component analysis; RF, random forest; T, tumor; N, node; LN, lymph node; LNM, lymph node metastasis; LM, liver metastasis; SLM, synchronous liver metastasis; DM, distant metastasis; SDM, synchronous distant metastasis; 5y DFS, 5-year disease-free survival; 3y DMFS, 3-year distant metastasis-free survival.

**Table 3:** Summary of high-risk clinical pathological factor prediction MRI radiomics-based studies.

First Author, Year	Study Design	MRI Time	Image Modality	Segmentation	Feature Selection Model	Endpoint	Refs.
Zhao *et al.*, 2021	R/S	Pre	T1, T2, PD	ROI	mRMR, LASSO	EMVI	[23]
Shu *et al.*, 2022	R/S	Pre	T2WI, T1WI, DWI, CE-T1WI	VOI	Bayes	EMVI	[24]
Wang *et al.*, 2023	R/M	Pre	T2WI, DWI, CE-T1WI	ROI, VOI	LASSO, mRMR	PFI	[25]
Wang *et al.*, 2025	R/S	Pre	T2WI, DWI	ROI	LASSO	PNI	[26]
Xu *et al.*, 2025	R/S	Pre	T2WI, DWI	ROI	SCC, LASSO	LVI	[27]
Ning *et al.*, 2025	R/M	Pre	T2WI, DWI	ROI	mRMR, LASSO	LVI	[28]
Liang *et al.*, 2024	R/S	Pre	T1WI, T2WI, DWI, CE-T1WI	VOI	ANOVA, correlation analysis, GBDT	CRM	[29]
Corines *et al.*, 2025	R/M	Pre	T2WI	VOI	SCC, RE	acellular mucin, cellular mucin	[30]
Li *et al.*, 2022	R/M	Pre	T2WI, CE-T1WI, DWI	VOI	variance threshold, Select-K-best, LASSO	TB	[31]
Qu *et al.*, 2023	R/M	Pre	T2WI	ROI	LASSO	TB	[34]
Peng *et al.*, 2024	R/S	Pre	T2WI, DWI, CE-T1WI	VOI	PCC, ANOVA	TB	[33]
Liu *et al.*, 2025	R/M	Pre	T2WI, DWI, CE-T1WI	ROI	SCC, RE	TB	[32]

**Abbreviations:** R, retrospective study; S, single-centre study; M, multicentre study; Pre, pretreatment; PD, proton density maps; T1WI, T1-weighted imaging; T2WI, T2-weighted imaging; DWI, diffusion-weighted imaging; CE-T1WI, contrast-enhanced T1-weighted imaging; ROI, region of interest; VOI, volume of interest; mRMR, minimum redundancy maximum relevance; LASSO, least absolute shrinkage and selection operator; SCC, spearman correlation coefficient; PCC, pearson correlation coefficient; ANOVA, analysis of variance; GBDT, gradient boosting decision tree; RE, recursive elimination; Select-K-best, select the K best features; EMVI, extramural vascular invasion; PFI, peripheral fat infiltration; PNI, perineural invasion; LVI, lymphovascular invasion; CRM, circumferential resection margin; TB, tumor budding.

**Table 4:** Summary of genetic markers prediction in MRI radiomics-based studies.

First Author, Year	Study Design	MRI Time	Image Modality	Segmentation	Feature Selection Model	Endpoint	Refs.
Cui *et al.*, 2020	R/M	Pre	T2WI	ROI	Univariate statistical tests, LR, DT, SVM	KRAS	[40]
Guo *et al.*, 2020	R/S	Pre	T1WI, T2WI, CE-T1WI	ROI	SVM	KRAS	[41]
Zhang *et al.*, 2021	R/S	Pre	T2WI	ROI	LASSO	KRAS	[8]
Meng *et al.*, 2019	R/S	Pre	T2WI	VOI	mRMR, WLCX	LN, tumor differentiation, Ki-67, HER-2, KRAS-2	[42]
Xiang *et al.*, 2023	R/M	Pre	T2WI	ROI	LASSO, SVM	KRAS	[44]
Cai *et al.*, 2024	R/M	Pre	T2WI, T1WI, DWI, CE-T1WI	ROI	PCA, LASSO	KRAS	[47]
Yao *et al.*, 2025	R/M	Pre	T2WI, DWI, CE-T1WI	ROI	mRMR, LASSO	MSI	[51]
Jing *et al.*, 2022	R/M	Pre	T2WI, DWI, CE-T1WI	ROI, VOI	LASSO	MSI	[48]
Li *et al.*, 2023	R/M	Pre	T1WI, T2WI, DWI, CE-T1WI	ROI	RF	MSI	[49]
Zhang *et al.*, 2023	R/S	Pre	T2WI, T1WI, ADC, CE-T1WI	VOI	ANOVA, correlation test, univariate logistic analysis, GBDT	MSI	[52]
Xing *et al.*, 2024	R/S	Pre	T2WI, T1WI, DWI, CE-T1WI	VOI	Multifactor logistic regression	MSI	[50]

**Abbreviations:** R, retrospective study; M, multicentre study; S, single-centre study; Pre, pretreatment; T1WI, T1-weighted imaging; T2WI, T2-weighted imaging; CE-T1WI, contrast-enhanced T1-weighted imaging; DWI, diffusion-weighted imaging; ADC, apparent diffusion coefficient; ROI, region of interest; VOI, volume of interest; LR, logistic regression; DT, decision tree; SVM, support vector machine; LASSO, least absolute shrinkage and selection operator; mRMR, minimum redundancy maximum relevance; WLCX, wilcoxon rank-sum test; PCA, principal component analysis; RF, random forest; ANOVA, analysis of variance; GBDT, gradient boosting decision tree; LN, lymph node; Ki-67, fraction of Ki-67-positive tumor cells; HER-2, human epidermal growth factor receptor 2; MSI, microsatellite instability.

**Table 5:** Summary of efficacy and response to neoadjuvant therapy evaluation MRI radiomics-based studies.

First Author, Year	Study Design	MRI Time	Image Modality	Segmentation	Feature Selection Model	Endpoint	Refs.
Nie *et al.*, 2016	R/S	Pre	T1WI, T2WI, DWI, DCE	ROI	ANN	pCR, GR	[58]
Yi *et al.*, 2019	R/S	Pre	T2WI	ROI	LASSO	pCR, GR, down-staging	[79]
Cheng *et al.*, 2021	R/S	Pre	T1WI, T2WI, T2FS	ROI, VOI	LASSO	pCR, GR	[61]
Homsi *et al.*, 2024	R/M	Pre	T1WI, T2WI, DWI	VOI	PCA	pCR	[64]
Wan *et al.*, 2021	R/S	Pre, Post	T2WI, DWI	ROI	LASSO	pCR	[74]
Xiang *et al.*, 2023	R/M	Pre	T2WI	ROI	PCA, LASSO	ypT0-2, treatment response	[77]
Cui *et al.*, 2019	R/S	Pre	T2WI, CE-T1WI, ADC	ROI	LASSO	pCR	[62]
Shaish *et al.*, 2020	R/M	Pre	T2WI	ROI	RFE	pCR, TRG, NAR	[70]
Pang *et al.*, 2021	R/M	Post	T2WI	ROI	LASSO	pCR	[69]
Li *et al.*, 2021	R/S	Pre, Post	T2WI	VOI	LASSO, PCA	pCR, TRG	[66]
Chen *et al.*, 2020	R/S	Pre, Post	T2WI	ROI	SCC, WLCX	pCR, GR, T down-staging	[60]
Wan *et al.*, 2022	R/S	Pre, Post	T2WI, DWI	ROI	mRMR, LASSO	pGR, ypT0-1N0	[73]
Shin *et al.*, 2022	R/S	Post	T2WI, ADC	VOI	LASSO	pCR	[71]
Zhang *et al.*, 2022	R/S	Pre	T2WI, DWI	ROI	LASSO	pCR	[80]
Lu *et al.*, 2024	R/S	Pre, Post	T2WI	ROI	LASSO	pCR	[67]
Tian *et al.*, 2025	R/M	Pre, Post	T2WI, DWI	GTV	LASSO, PCA	pCR	[72]
Palmisano *et al.*, 2020	R/S	Pre, Mid, Post	T2WI, DWI, ADC	ROI, VOI	SCC	CR, PR, NR	[68]
Wang *et al.*, 2024	R/M	Pre	T1WI, T2WI	ROI	LASSO	cCR, pCR	[76]
Bulens *et al.*, 2020	R/S	Pre, Post	T2WI, DWI	ROI	LASSO, PCA	ypT0–1N0	[7]
Pizzi *et al.*, 2021	R/S	Pre	T2WI	ROI	PLS	treatment response	[63]
Wang *et al.*, 2022	R/S	Pre	T2WI, DWI, CE-T1WI	VOI	mRMR, LASSO	treatment response	[75]
Chen *et al.*, 2022	P/S	Pre	T2WI	ROI	PCC, univariate analysis, LASSO	GR	[59]
Yardimci *et al.*, 2023	R/S	Pre	T2WI	VOI	RA, PWCA, ANOVA, RFE, Kruskal-Wallis, Relief	treatment response	[78]
Jiang *et al.*, 2023	R/S	Pre	T2WI, DWI	ROI	variance threshold, Select-K-best, LASSO	treatment response	[65]
Zhu *et al.*, 2024	R/M	Pre, Post	T2WI, DWI	ROI	RFE	treatment response	[81]

**Abbreviations:** R, retrospective study; S, single-centre study; M, multicentre study; P, prospective study; Pre, pretreatment; Post, post-treatment; Mid, midtreatment; T1WI, T1-weighted imaging; T2WI, T2-weighted imaging; DWI, diffusion-weighted imaging; DCE, dynamic contrast-enhanced; T2FS, T2-weighted fatsuppression; CE-T1WI, contrast-enhanced T1-weighted imaging; ADC, apparent diffusion coefficient; ROI, region of interest; VOI, volume of interest; GTV, gross tumor volume; ANN, artificial neural network; LASSO, least absolute shrinkage and selection operator; PCA, principal component analysis; RFE, recursive feature elimination; SCC, spearman correlation coefficient; PCC, pearson correlation coefficient; WLCX, wilcoxon rank-sum test; mRMR, minimum redundancy maximum relevance; PLS, partial least square; RA, reliability analysis; PWCA, pair-wise correlation analysis; ANOVA, analysis of variance; RFE, recursive feature elimination; Kruskal-Wallis, Kruskal-Wallis H test; Relief, relief algorithm; Select-K-best, select the K best features; pCR, pathological complete response; pGR, pathological good response; cCR, clinical complete response; GR, good response; NAR, neoadjuvant rectal score; TRG, tumor regression grade; CR, complete responders; PR, partial responders; NR, non-responders; ypT0-2, post-treatment pathological tumor stage 0 to 2; ypT0-1N0, post-treatment pathological tumor stage 0 to 1 with no regional lymph node metastasis.

**Table 6:** Summary of recurrence, prognosis, and survival analysis of MRI radiomics-based studies.

First Author, Year	Study Design	MRI Time	Image Modality	Segmentation	Feature Selection Model	Endpoint	Refs.
Guo *et al.*, 2025	R/S	Pre	T1WI, T2WI, CE-T1WI, HR-T2WI	ROI, VOI	LR, SVM, KNN, RF, ET, XGBoost, LGBM	OS	[89]
Fu *et al.*, 2025	R/M	Pre	T2WI, DWI	VOI	LASSO, COX regression	DFS	[88]
Cui *et al.*, 2021	R/S	Pre	T2WI, CE-T1WI, ADC	VOI	Boruta algorithm	DFS	[86]
Cui *et al.*, 2022	R/S	Pre	T2WI, DKI, CE-T1WI	VOI	feature stability, Cox PH	DFS	[85]
Li *et al.*, 2023	P/M	Pre	T2WI	VOI	LASSO	3y RFS, LNM	[93]
Li *et al.*, 2023	P/M	Pre	T2WI	VOI	LASSO	3y RFS, TD	[35]
Li *et al.*, 2024	P/S	Pre	T2WI	VOI	LASSO	3y RFS, ENE	[92]
Knuth *et al.*, 2024	R/S	Pre	T2WI, ADC, K-trans, BF	VOI	ICC	5y PFS, OS	[91]
Zhao *et al.*, 2024	R/S	Pre	T2WI	VOI	LASSO, Cox analysis	5y DMFS	[96]
Huang *et al.*, 2024	R/M	Pre	T2WI, DWI, CE-T1WI	ROI	LASSO, T-test	3y DFS	[90]
Xie *et al.*, 2024	R/M	Pre	T2WI, DWI	ROI	ICC, SCC, Cox analysis	DFS	[94]
Yao *et al.*, 2024	R/M	Pre	T2WI, DWI, CE-T1WI	ROI	mRMR, LASSO	DFS	[95]
Fan *et al.*, 2025	R/M	Pre	T2WI	VOI	mRMR, LASSO	3y DFS	[87]
Chen *et al.*, 2021	R/S	Post	T2WI, DWI, CE-T1WI, VIBE	VOI	PCA	local recurrence	[84]

**Abbreviations:** R, retrospective study; S, single-centre study; M, multicentre study; P, prospective study; Pre, pretreatment; Post, post-treatment; T1WI, T1-weighted imaging; T2WI, T2-weighted imaging; CE-T1WI, contrast-enhanced T1-weighted imaging; HR-T2WI, high-resolution T2-weighted imaging; DWI, diffusion-weighted imaging; ADC, apparent diffusion coefficient; DKI, diffusion kurtosis imaging; K-trans, volume transfer constant; BF, blood flow; VIBE, volume interpolated body examination; ROI, region of interest; VOI, volume of interest; LR, logistic regression; SVM, support vector machine; KNN, K-nearest neighbour; RF, random forest; ET, extremely randomized tree; XGBoost, extreme gradient boosting; LGBM, light gradient boosting machine; LASSO, least absolute shrinkage and selection operator; Cox PH, Cox proportional hazards; ICC, intraclass correlation coefficient; SCC, spearman correlation coefficient; mRMR, minimum redundancy maximum relevance; PCA, principal component analysis; OS, overall survival; DFS, disease-free survival; 3y DFS, 3-year disease-free survival; 5y DMFS, 5-year distant metastasis-free survival; 3y RFS, 3-year recurrence-free survival; 5y PFS, 5-year progression-free survival; LNM, lymph node metastasis; TD, tumor deposit; ENE, extranodal extension.

## LIST OF ABBREVIATIONS

RC: Rectal cancer
MRI: Magnetic resonance imaging
CRC: Colorectal cancer
T2WI: T2-weighted imaging
DWI: Diffusion-weighted imaging
DCE: Dynamic contrast-enhanced
ROI: Region of interest
GLCM: Gray-level co-occurrence matrix
GLRLM: Gray-level run-length matrix
LoG: Laplacian of Gaussian
LASSO: Least absolute shrinkage and selection operator
PCA: Principal component analysis
SVM: Support vector machine
RF: Random forest
CT: Computed tomography
mp-MRI: Multi-parametric magnetic resonance imaging
T1WI: T1-weighted imaging
TNM: Tumor-node-metastasis
nCRT: Neoadjuvant chemoradiotherapy
TME: Total mesorectal excision
AUC: Area under the curve
LNM: Lymph node metastasis
CE-T1WI: Contrast-enhanced T1-weighted imaging
SLM: Synchronous liver metastasis
SDM: Synchronous distant metastasis
KRAS: Kirsten rat sarcoma viral oncogene homolog
EMVI: Extramural vascular invasion
PFI: Peripheral fat infiltration
PNI: Perineural invasion
LVI: Lymphovascular invasion
CRM: Circumferential resection margin
TB: Tumor budding
MSI: Microsatellite instability
EGFR: Epidermal growth factor receptor
LARC: Locally advanced rectal cancer
pCR: Pathological complete response
NAR: Neoadjuvant rectal (score)
MMR: Mismatch repair
MLH1: MutL Homolog 1
GR: Good response
CEA: Carcinoembryonic antigen
OS: Overall survival
DFS: Disease-free survival
RS: Radiomics signature
C-index: Concordance index
VOI: Volume of interest
CDSS: Clinical Decision Support System
PACS: Picture Archiving and Communication System
